# Dynamic 4 dimensional contrast enhanced MRI for localization in primary hyperparathyroidism

**DOI:** 10.1007/s12020-025-04239-2

**Published:** 2025-04-22

**Authors:** Sabri Engin Altintop, Mahi Nur Cerit, Emetullah Cindil, Halit Nahit Sendur, Tugba Barlas, Mehmet Muhittin Yalcin, Alev Eroglu Altinova, Mujde Akturk, Fusun Balos Toruner, Mehmet Ayhan Karakoc, Ethem Turgay Cerit

**Affiliations:** 1 Gazi University Faculty of Medicine, Department of Endocrinology and Metabolism, Ankara, Turkey; 2 Gazi University Faculty of Medicine, Department of Radiology, Ankara, Turkey

**Keywords:** Primary hyperparathyroidism, Parathyroid adenoma, Ultrasonography, Tc 99m sestamibi SPECT scan, 4D MRI

## Abstract

**Purpose:**

It can be challenging to localize the lesions in certain cases of primary hyperparathyroidism. Recently, it has been proposed that assessing the localization of parathyroid lesions with dynamic images enhances the diagnostic power of standard MRI (magnetic resonance imaging) due to the hypervascular structure of these lesions. In this study, we aimed to evaluate the success of four-dimensional dynamic perfusion MRI (4D MRI) in localizing parathyroid lesions.

**Methods:**

Thirty patients who underwent 4-dimensional dynamic MRI diagnosed with primary hyperparathyroidism and indications for surgery, whose USG (ultrasonography) and/or Tc 99m sestamibi SPECT scan were negative or discordant, were included. The sensitivity and positive predictive values (PPV) were calculated for each imaging modality.

**Results:**

Of the 30 patients, 29 had parathyroid adenoma, and one had parathyroid hyperplasia in histopathologic examination. 4D MRI accurately identified the location of parathyroid lesions in 25 of 30 patients (sensitivity 83.3%, PPV 96.1%), whereas USG successfully identified the lesion location in 21 patients (sensitivity 70%, PPV 91.3%) and Tc 99m sestamibi SPECT scan in 17 patients (sensitivity 56.7%, PPV 94.4%). The sensitivity of the combination of three imaging modalities was found to be 96.7%.

**Conclusion:**

4D MRI can be utilized as a complementary imaging modality to localize parathyroid lesions, offering the advantage of no ionizing radiation, especially when USG and/or Tc 99m sestamibi SPECT scans cannot reliably identify them.

## Introductıon

Primary hyperparathyroidism (PHPT) is an endocrine disease resulting from excess release of parathyroid hormone (PTH) from one or more of the parathyroid glands. It is often caused by a single adenoma (80%), although it can also result from hyperplasia (15%), double adenomas (2–4%), or parathyroid cancer (1%) [1]. Accurate preoperative localization of abnormal parathyroid tissue is crucial for allowing parathyroid surgery to be performed with a minimally invasive method [2].

Neck ultrasonography (USG) and Tc99m sestamibi SPECT scans are commonly employed as first-line imaging techniques to locate parathyroid glands. The diagnostic performance of ultrasound may vary because of the operator-dependent nature of the modality Ultrasound has a 48–96% sensitivity (pooled sensitivity 76%) for detecting aberrant parathyroid glands [3]. The success rate of USG decreases in patients with smaller parathyroid glands or ectopic and mediastinal glands and also in obese patients [4]. Sestamibi scanning findings may frequently be inconclusive in patients with parathyroid hyperplasia, multiglandular diseases, and concurrent thyroid nodules [5]. Furthermore, in 12–25% of individuals, the Tc99m sestamibi SPECT scan can be negative [6].

There is no consensus on which imaging modality should be used initially in cases where USG and Tc 99m sestamibi SPECT scans fail to locate the parathyroid glands [7]. Other imaging modalities that can be utilized in this circumstance include parathyroid four-dimensional computed tomography (4D-CT), magnetic resonance imaging (MRI), 11C-methionine positron emission tomography (MET-PET-CT scan), and 18F-fluorocholine positron emission tomography (18F-fluorocholine PET). Each method has its own advantages and disadvantages [8].

4D-CT has become increasingly common for presurgical planning of PHPT due to its superior diagnostic performance compared to USG and Tc 99m sestamibi SPECT scans [9]. In a study of 45 patients with prior neck exploration, 4D-CT demonstrated an 88 percent sensitivity for the identification of aberrant parathyroid glands, compared to neck USG or Tc 99m sestamibi SPECT scan (54 and 21 percent, respectively) [10]. However, 4D-CT can result in significant radiation doses ranging from 5.56–10.4 mSv, based on the number of phases scanned. Since thyroid cancer is mostly caused by radiation exposure, especially in younger patients, 4D-CT should be used with caution [2, 11].

MET-PET-CT (11C-methionine positron emission tomography and computed tomography combined), which utilizes 11C-methionine as a radiotracer to locate the aberrant parathyroid glands, has a sensitivity of 86% and a PPV of 93% [12]. Another modality that has been effectively utilized to locate parathyroid adenomas is 18-fluorocholine-PET-CT, which has a sensitivity ranging from 80 to 100 percent [13].

4D dynamic contrast-enhanced MR (4D MRI) imaging is an imaging method that benefits from the hypervascular character of aberrant parathyroid lesions [14]. The identification of ectopic lesions more accurately and the differentiation of thyroid nodules, cervical lymph nodes, and fat tissue from parathyroid glands are two substantial advantages of 4D sectional imaging techniques in parathyroid disorders [15]. The addition of dynamic imaging to conventional MRI significantly increases the detection rate of parathyroid adenomas [16, 17]. Performing 3-Tesla MRI with an added “4D” (time-resolved enhancement kinetics) provides a sensitivity of over 90%, outperforming ultrasound (76%) and Tc 99m sestamibi SPECT scan (71%). Furthermore, the sensitivity of combining all three modalities (USG, Tc 99m sestamibi SPECT scan, and 4D-MRI) may reach 100% [18]. Four-dimensional MRI demonstrated 85% accuracy in differentiating single gland disease from multiglandular diseases, with a success rate of 74% for laterality and 77% for quadrant localization [19]. Furthermore, 4D MRI has demonstrated the ability to accurately detect “double adenomas,” a known risk factor for recurrent PHPT. In addition, due to its superior soft tissue resolution and the water-fat separation techniques, MRI offers superior anatomic information and might be more effective in detecting small parathyroid gland lesions [8]. The main advantage of MRI is that it does not require the use of ionizing radiation [20].

Despite all of the advances in imaging technology, 2–10% of patients will still have recurrent or persistent disease after surgery [21]. Therefore, studies continue to establish the optimal imaging method. 4D MRI investigations have yielded positive results in recent years.

The aim of this study was to investigate the diagnostic accuracy of 4D MRI at 3 Tesla for the identification of aberrant parathyroid glands in combination with USG, Tc 99m sestamibi SPECT scan, and surgical and pathological findings.

## Material and methods

This retrospective study was approved by the Clinical Studies Ethical Committee of Gazi University Faculty of Medicine.

### Characteristics of patients

Patients who were diagnosed with primary hyperparathyroidism and had surgical indications and underwent dynamic 4-dimensional contrast-enhanced MRI between January 2018 and July 2023 were included in the study. 4D MRI had been performed when no lesion was revealed by neither Tc 99m sestamibi SPECT scan nor USG or when the results of two imaging methods were discordant with each other.

**Fig. 1:**
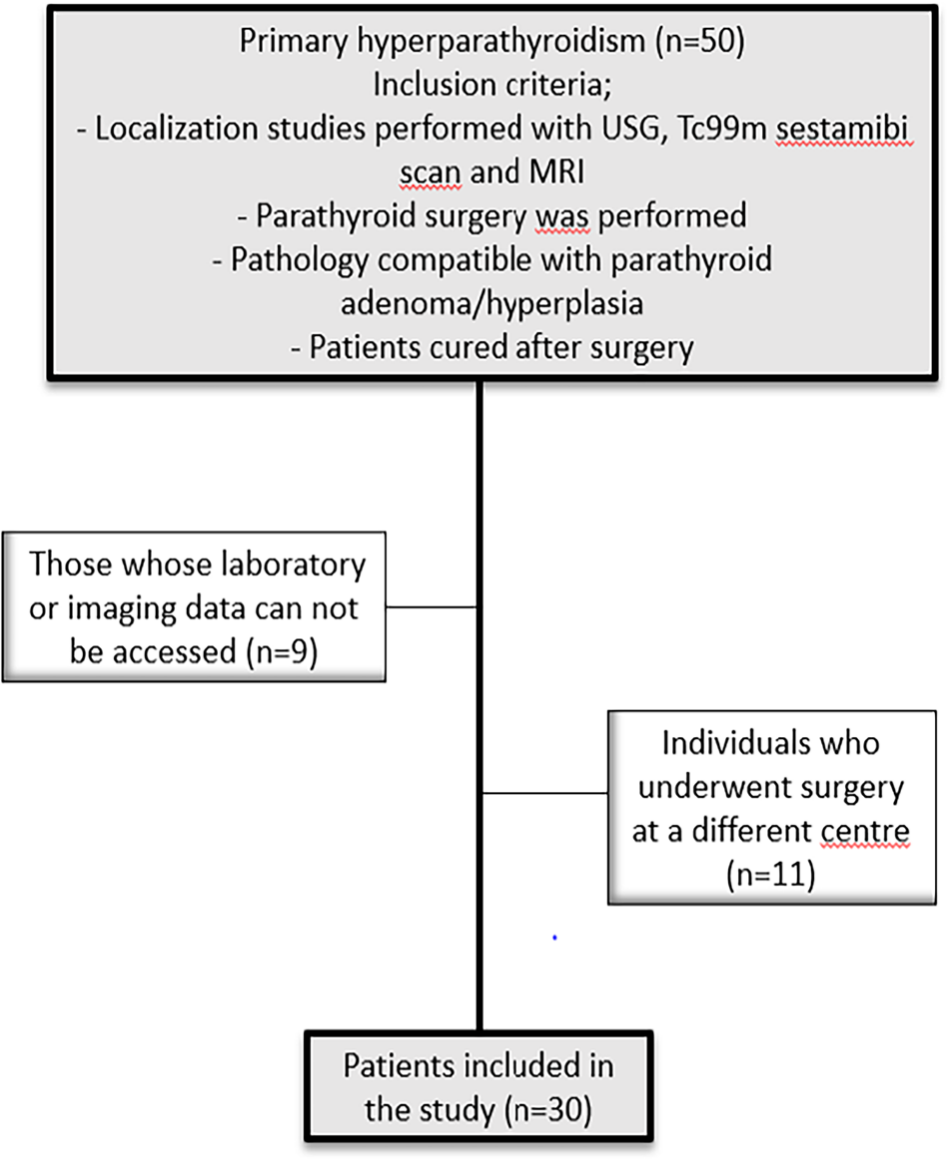
Flow diagram of inclusion and exclusion criteria. USG ultrasonography, MRI magnetic resonance imaging

The inclusion and exclusion criteria for the study are given in Fig. 1 below.

The age and gender of the patients were recorded. The following criteria for parathyroid surgery (Age <50 years, serum calcium >1 mg/dl (0.25 mmol/L) above the upper limit of normal, presence of nephrolithiasis or nephrocalcinosis, high urinary calcium, presence of osteoporosis, glomerular filtration rate (GFR) < 60 ml/min/1,73 m^2^) were acquired from the patients’ previous electronic medical data. The serum parathormone (PTH), calcium, phosphorus, albumin, GFR, and 25-OH vitamin D values of the patients in the preoperative and postoperative first six months were also noted. If calcium and/or PTH levels remained normal during the postoperative 6-month follow-up, the patient was considered cured. The patients who did not fulfill these criteria were not included in the study.

### Neck ultrasonography (USG)

Ultrasonography reports of all patients were accessible through the hospital’s electronic database. The four-quadrant system (right superior-inferior and left superior-inferior), which is based on lesion localization in relation to the vertical midline and a transverse plane through the middle of the thyroid gland, was used to determine the localization of the parathyroid gland. USG examinations for all patients were performed utilizing the G.E. Logiq P5 ultrasound device. The examination was performed with the patient’s neck hyperextended, using both transverse and longitudinal imaging from the hyoid bone (cranially) to the thoracic inlet (caudally) and extending to the carotid arteries laterally. A parathyroid lesion was considered when a typical hypoechoic lesion with a peripheral vascular arc or a prominent polar feeding vessel was found [22].

### Tc 99m sestamibi SPECT scan

99mTc sestamibi scan reports of all patients were accessible through the hospital’s electronic database. Sestamibi scintigraphy with single photon emission computed tomography (SPECT) was also performed on all patients at our institution. Early and delayed-phase images were typically acquired following the injection of 99 mTc sestamibi to detect areas of retained radiotracer activity. Distinguishing between thyroid and parathyroid glands on delayed images is possible due to the different retention of the radiotracer technetium-99m sestamibi (MIBI). Hyperfunctioning parathyroid tissues, characterized by increased mitochondrial activity, exhibit prolonged retention of the radiotracer, whereas thyroid tissue demonstrates a faster washout pattern [23, 24].

### 4D dynamic perfusion MRI

#### Image acquisition

The parathyroid MR images of the patients included in the study, taken between 2018 and 2023, were obtained using a 3T MRI machine (Magnetom Verio syngo MR B17, Siemens Healthcare, Erlangen, Germany) with the patients breathing freely. All image analyses were performed using Siemens Healthineers Syngo via VB30 workstation. A combination 12-element head and neck coil was used for radiofrequency signal reception.

Time-Resolved Angiography with Stochastic Trajectories sequence, one of the fast imaging tools, and improved parallel imaging techniques such as Generalized Autocalibrating Partially Parallel Acquisition (GRAPPA) was chosen to observe dynamic changes after contrast injection with high temporal and spatial resolution. MRI protocols are presented in Table 1 and the critical technical details of TWİST sequence are presented in Table 2. The protocol parameters were optimized to provide high acceleration, clear image quality, and good temporal resolution, thus allowing rapid and clear visualization of the arterial phase.

**Table 1:** 4D MRI ımaging protocol

Sequence	TR/TE (ms)	Matrix	FOV (mm)	Voxel size (mm³)	Gap (mm)	TA (min)
2D protocol						
Sag T2 TSE	3770/109	288 × 384	200	0.9 × 0.6 × 5	0.6	1:36
T2 Cor STIR	5300/93	320 × 320	240	0.8 × 0.8 × 5	1.5	2:14
Cor T2 fs	3660/101	307 × 384	200	0.8 × 0.6 × 3	0.6	4:47
Ax T2 fs	3500/91	230 × 384	180	0.8 × 0.5 × 4	0.6	2:43
Ax T1	800/10	230 × 384	200	0.5 × 0.5 × 4	0.6	1:30
Sag T1 fs	607/10	269 × 384	200	0.5 × 0.5 × 3	0.6	2:28
Cor T1 fs	650/10	192 × 320	240	1.3 × 0.8 × 5	0.6	1:57
Cor T1	727/12	256 × 320	200	0.8 × 0.6 × 3	0.6	3:32
4D protocol						
TWIST	4.89/1.89	138 × 192	240	1.7 × 1.3 × 2.4	—	7:33

*TR / TE* repetition time / echo time, *FOV* field of view, *TA* time of acquisition, *Sag* sagittal, *TSE* turbo spin echo, *Cor* coronal, *Ax* axial, fs: fat suppressed, *TWIST* time-resolved imaging with stochastic trajectories

**Table 2:** Technical details of TWIST (Time-resolved Angiography with Stochastic Trajectories) sequence

Sequence type	TWIST
Voxel size (Image size)	1.7 × 1.3 × 2.4 mm^3^
Temporal resolution	6.75 s
Total number of phases	64
Acquisition time (TA)	7 min 25 s
Image matrix	192 base resolution, 72% phase resolution, slice resolution 60%
Flip angle	12°
TR (Repetition Time)	4.89 ms
TE (Echo Time)	1.89 ms
Bandwidth	260 Hz/Px
Sampling density	21%
TWIST central region ratio (Central region A)	51%
Parallel imaging technique	GRAPPA (Generalized Autocalibrating Partially Parallel Acquisition)
GRAPPA acceleration factor	2
Slab thickness	76 mm
Slice thickness	2.4 mm
Distortion correction	2D (active)

T1-weighted (T1W) sequences were acquired before and after, whereas TWIST sequences were acquired only after intravenous injection of gadolinium contrast medium 0.1 mmol/kg body weight (gadoterate meglumine; Dotarem, Guerbet, Villepinte, France). Sixty-four temporal frames were obtained during 453 s of acquisition time after intravenous injection of Dotarem at 4 mL/s, followed by 20 mL of saline at 4 mL/s.

#### Image analysis

A single experienced radiologist with over 10 years of expertise in interpreting MR imaging for parathyroid adenomas evaluated all imaging datasets. The radiologist was blinded to patient histories, histopathological findings, and the results of other imaging modalities to minimize bias.

Reader searched the most common locations of the parathyroid glands (native) as well as all possible ectopic locations [25]. For each gland, the side was recorded in relation to the midline, and the quadrant was recorded as superior or inferior, depending on its relation to the midpole of the thyroid gland.

Since the majority of PTAs are T2 hyperintense and show early arterial enhancement, T2w fat-sat and TWIST sequences were the most frequently evaluated sequences for preoperative localization. Having high spatial resolution voxels (1.7 × 1.3 × 2.4 mm^3^) from the mandibular rim to the manubrium allowed for accurate examination of both native and ectopic adenomas as small as 3 mm. Having this data set within seconds provided a true 4D capability for demonstration of the contrast-enhancement curve of PTA over the course of image acquisition (445 s). By using the TWIST sequence, 64 time slices were acquired every 6.75 s, and the hypervascular nature of these lesions was revealed accurately [19, 26].

Abnormal parathyroid glands were identified as arterial-enhancing T2 hyperintense nodules in the native parathyroid gland space or along the expected embryologic course of parathyroid glands, such as the tracheoesophageal groove or superior mediastinum. In addition, they were detected based on the following standardized criteria [27].

1. **Early enhancement:** Nodules showing arterial phase enhancement, with signal intensity peaking within 1–2 frames of carotid artery enhancement onset.
2. **Venous washout:** Nodules exhibiting maximal signal intensity during the early venous phase, followed by a marked decrease in signal intensity in the late venous phase.
3. **Morphological characteristics:** Lesions were assessed for their size, shape (e.g., oval or round), and border distinctness relative to adjacent thyroid or other tissues.

**Fig. 2:**
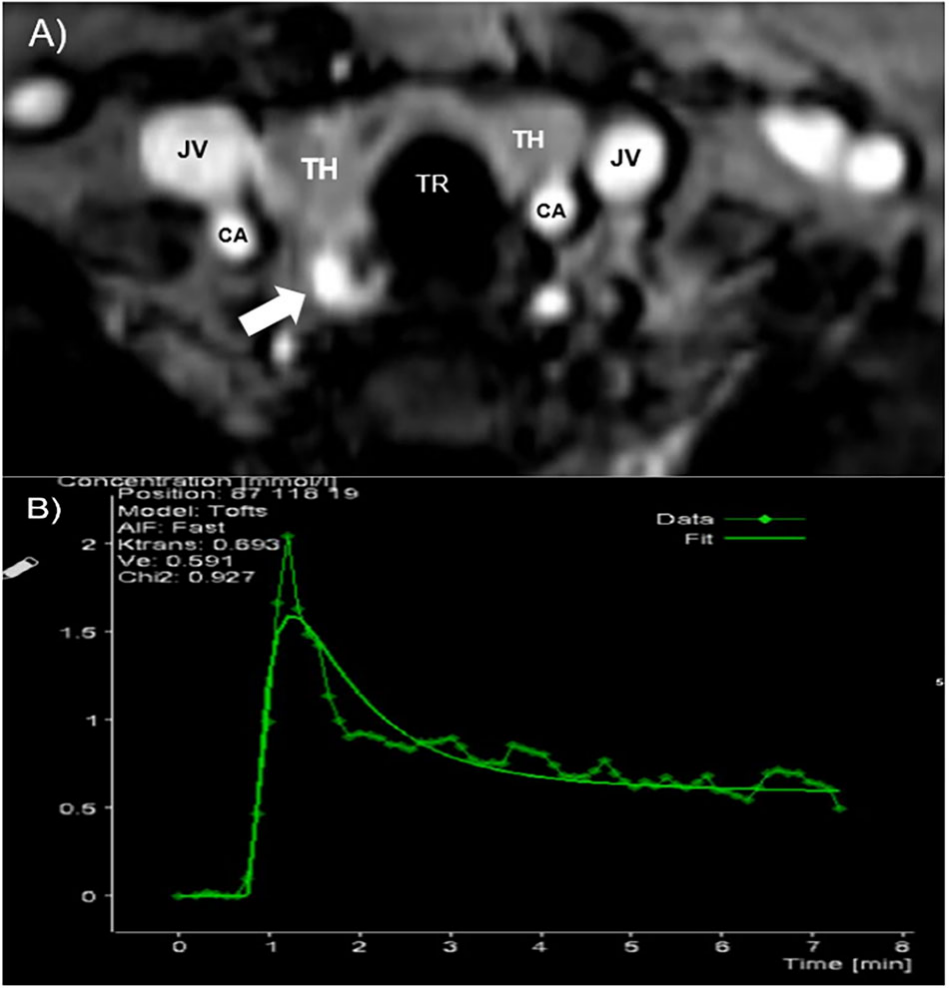
MR image of a 32-year-old man with PHPT. **A** Axial T1 TWIST sequence demonstrates a hyperintense lesion in the right inferior quadrant (JV jugular vein, CA carotis artery, TH thyroid lobes, TR trachea). **B** Dynamic time-contrast concentration curve of a parathyroid adenoma

For each lesion, dynamic signal intensity (SI) patterns were quantitatively analyzed across the arterial, early venous, and late venous phases by constructing a contrast concentration-time curve. These SI measurements were used to differentiate parathyroid adenomas from thyroid tissue and lymph nodes. The analysis focused on detecting specific enhancement patterns, including early enhancement, peak SI, and contrast washout rates. This is particularly useful when you have several PTA candidate lesions to narrow the differential diagnosis and provide the surgeon with the lesion most likely to be a true PTA. PTAs show significantly more rapid arterial enhancement than thyroid tissue and normal cervical lymph nodes [14] (Fig. 2).

### Parathyroid surgery and histopathological assesment

Detailed surgical and pathology reports of all patients, which were accessible via the hospital’s electronic database, were thoroughly reviewed. Surgery of the patients was performed by two general surgeons experienced in parathyroid and thyroid surgery. Minimally invasive parathyroid surgery or bilateral explorative neck surgery was performed based on preoperative USG, sestamibi scan, and 4D MRI results. Bilateral neck exploration was performed, especially in cases in which imaging results are discordant and also in one patient with parathyroid hyperplasia. The operation was considered successful and completed if the excised lesion was diagnosed as a parathyroid gland on a frozen section and the serum intact PTH level reduced more than 50% of the preoperative level. However, the definitive diagnosis was established after detailed histopathological examinations.

### Statistical analysis

The statistical analysis of this study was performed using the SPSS 22.0 software program. P values of <0.05 were considered significant. The normality assumption was examined by using the Shapiro-Wilk test. Descriptive statistics were given as mean and standard deviation (mean ± SD) for parametric variables, while non-parametric variables were expressed by using median and IQR (interquartile range). In addition, categorical variables were expressed as percentages (%). A Wilcoxon signed-rank test was used for dependent variable analysis. A chi-square test was utilized to compare different categorical variables. Spearman correlation analysis was performed to evaluate the relationship between the numerical variables. Sensitivity and positive predictive values (PPV) were calculated for each imaging modality. Specificity was not assessed because all patients were required to have a postoperative diagnosis of parathyroid adenoma or hyperplasia.

## Results

30 patients were included in the study (23 female and 7 male). The mean age of all patients was 55.6 ± 12.4. Patients categorization based on their surgical indication is illustrated in Table 3 below.

**Table 3:** Patient categorization according to surgical indications of PHPT

	Patients (*n* = 30)
Age < 50 years, n (%)	10 (33.3)
Presence of nephrolithiasis/nephrocalcinosis, n (%)	6 (20.0)
Serum calcium >1 mg/dl above ULN, n (%)	12 (40.0)
High urinary calcium, n (%)	12 (40.0)
Presence of osteoporosis, n (%)	7 (23.3)
GFR < 60 ml/min/1.73 m^2^, n (%)	3 (10.0)

*ULN* upper limit of normal, *GFR* glomerular filtration rate

Of the 30 patients, all had parathyroid adenoma, except one who had hyperplasia in four parathyroid glands based on histopathologic examination. According to surgical results, 15 lesions were situated in the lower right quadrant, 13 in the lower left quadrant, 2 in the upper right quadrant, 2 in the upper left quadrant, and 1 at the left posterior of the manubrium sterni.

The average largest diameter of lesions examined by 4D MRI was 12.4 ± 8.6 mm, while the average largest diameter of lesions removed by surgery was 14.9 ± 10.6. A strong correlation was determined between measurements made by MRI and histopathological examination (*p* < 0.001, r = 0.781).

Following the surgery, which was performed utilizing the combination of three imaging modalities (USG, Tc 99m sestamibi SPECT scan, and 4D MRI), it was observed that PTH and calcium levels significantly declined while phosphorus levels increased (*p* < 0,001, *p* < 0,001, and *p* = 0,001, respectively) (Table 4).

**Table 4:** Laboratory findings of the patients before and after the surgery

	Preoperative	Postoperative (0–6 months)	p value
PTH (pg/ml)	140.9 (108.8–206.2)	26.8 (10.2–55.1)	<0.001
Ca (mg/dl)	11.1 (11.0–11.6)	9.7 (9.0–9.8)	<0.001
P (mg/dl)	2.7 (2.1–3.2)	3,4 (3.0–3.8)	0.001
Albumin (g/dl)	4.41 ± 0.28	4.48 ± 0.93	0.101
25-OH vitamin D (ng/ml)	19.0 (14.9–26.2)	27.0 (20.0–31.5)	0.225

*PTH* parathyroid hormone, *Ca* calcium, *P* phosphorus, *pg/ml* picogram/milliliter), *mg/dl* miligram/desiliter, *g/dl* gram/desiliter, *ng/ml* nanogram/mililiter

The sensitivity of USG and Tc 99m sestamibi SPECT scan was 70.0% (21/30) and 56.7% (17/30), respectively. USG identified the parathyroid lesions (false positive) incorrectly in two patients, while it was unable to detect the lesions (false negative) in eight patients. On the other hand, Tc 99m sestamibi SPECT scan inaccurately located the parathyroid lesion in one patient (false positive) and failed to find parathyroid lesions (false negative) in 15 patient.

In 11 of 13 cases in which Tc 99m sestamibi SPECT scan failed to locate the lesions, USG accurately identified them. Conversely, in 9 cases missed by USG, Tc 99m sestamibi SPECT scan was able to find 7 of them. 4D MRI accurately found the parathyroid lesions in 25 patients (sensitivity 83.3%) while incorrectly localizing the parathyroid lesion in one patient (false positive). Additionally, there were four false-negative cases.

Sensitivity and positive predictive values of each imaging modality are given below (Table 5). A combination of three imaging modalities was unable to locate the parathyroid lesion in only one patient.

**Table 5:** Sensitivity and positive predictive values of imaging methods

Imaging Modality	Sensitivity n (%)	PPV n (%)
USG	21/30 (70.0)	21/23 (91.3)
Tc 99m sestamibi SPECT scan	17/30 (56.7)	17/18 (94.4)
4D MRI	25/30 (83.3)	25/26 (96.1)
USG + Tc 99m sestamibi SPECT scan + 4D MRI	29/30 (96.7)	30/33 (90.9)

*PPV* positive predictive value, *SPECT* Single-photon emission computed tomography, *USG* Ultrasonography, *4D MRI* Dynamic contrast enhanced four dimensional MRI

There are a few cases in which USG and/or Tc 99m sestamibi SPECT scan fail to identify the location of the lesions or the findings of these two imaging modalities are inconsistent with each other. The success of 4D MRI in these cases is given below (Table 6).

**Table 6:** Accurate Localization Rates of 4D MRI in Comparison to USG and Tc 99m Sestamibi SPECT Scan Results

USG/Tc 99m sestamibi SPECT scan result	Sensitivity n (%)	PPV n (%)
USG negative or FP	6/9 (66.7)	6/6 (100)
Tc 99m sestamibi scan negative or FP	10/13 (76.9)	10/11 (90.9)
USG and Tc 99m sestamibi scan negative	1/2 (50.0)	1/1 (100)
USG and Tc 99m sestamibi scan discordant	15/19 (78.9)	15/16 (93.75)

*FP* false positive

MRI successfully identified 6 of 21 parathyroid lesions when USG and Tc 99m sestamibi SPECT scan revealed both negative or inconsistent results (sensitivity 76.1% and PPV 94.1%).

When imaging modalities were examined for 15 lesions in the right inferior location, MRI successfully identified the lesion in 14 (93.3%), USG in 10 (66.6%), and Tc 99m sestamibi SPECT scan in 6 (40%). In the left inferior position of 13 lesions, MRI detected 10 (76.9%), USG 10 (76.9%), and Tc 99m sestamibi SPECT scan 9 (69.2%) with success.

## Discussion

In this study, we primarily focused on investigating the localization success of the 4D MRI in cases in which USG and Tc 99m sestamibi SPECT scans fail to locate the abnormal parathyroid glands. The sensitivity of 4D MRI (83.3%) was found to be considerably higher than that of USG (70.0%) and Tc 99m sestamibi SPECT scan (56.7%). 4D MRI was able to identify 78.9% of the lesions (15/19) in cases where USG and Tc 99m sestamibi SPECT scan results were incompatible. Moreover, with the combination of three imaging modalities, parathyroid lesions were accurately localized in 29 of 30 patients (96.7%).

There are a limited number of studies in the literature investigating the localization performance of 4D MRI in primary hyperparathyroidism. Ozturk et al. reported the sensitivity and positive predictive value of 4D MRI as 90.5 and 95.0%, respectively [26]. Grayev et al. demonstrated a lower sensitivity of MRI (64%) compared to USG (88%) and 99mTc-sestamibi (72%) [28]. This may be attributable to the fact that adenomas missed by MRI tend to be slightly smaller than those missed by the other two imaging methods. In a study conducted by Memeh et al., the sensitivity of 4D MRI was found to be 100% for single adenoma and 64% for multiglandular disease, while the overall sensitivity of 4D MRI was 84.64% [19]. In another study, Becker et al. determined the sensitivity of 4D MRI as 92% for single gland disease, while for multigland disease, it was 74% [2]. Lower sensitivity of 4D MRI in multiglandular disease might be explained by smaller glands being noted in patients with multiglandular disease. In our study, the sensitivity and PPV of 4D MRI were determined as 83.3% and 96.1%, respectively. Since only one patient had multiglandular disease, we were unable to assess the sensitivity of 4D MRI in multiglandular disease.

Ozturk et al. reported the sensitivity of the combination of three imaging modalities as 100% similar to our study (96.7%). A parathyroid lesion could not be localized only in one patient with three imaging modalities combined in our study. 18-fluorocholine PET-CT also yielded a negative result. Following that, the patient had bilateral explorative parathyroid surgery, during which the 8 mm lesion was identified in the right inferior region. The complexity of this case and small size of the lesion might explain the reason for not localizing when the three imaging modalities are used together.

In our study, the PPV of the combination of three imaging modality (87.7%) was determined to be lower than USG (91.3%), Tc 99m sestamibi SPECT scan (94.4%), and 4D MRI (96.1%) separately. The reason for this is that the number of false positive cases increases when three imaging modalities are combined compared to these three modalities alone.

MRI accurately identified 16 of 21 lesions in cases where USG and Tc 99m sestamibi SPECT scans yielded both negative or inconsistent results with each other (sensitivity %76.1, PPV %94.1) in this study, which was similar to the literature (sensitivity 82.4% and PPV 93.3%) [25].

Only one false-positive case was detected on 4D MRI in this study. 4D MRI revealed two distinct lesions in this case: one in the left inferior quadrant (true positive) and another in the right inferior quadrant (false positive). Following surgical exploration, pathological examination confirmed that the lesion in the left inferior quadrant was a parathyroid adenoma, while the lesion in the right inferior quadrant was a reactive lymph node. Lymph nodes may occasionally exhibit atypical enhancement patterns similar to those of parathyroid adenomas [29].

Neck ultrasonography and Tc99m sestamibi SPECT imaging are usually first-line imaging modalities to localize the parathyroid lesion causing PHPT [30]. It can be challenging to assess the sensitivity of USG as it is operator-dependent. A meta-analysis evaluating the performance of ultrasound demonstrated a sensitivity or detection rate of 76.1% and a PPV of 93.2% [3]. In our study, the sensitivity and positive predictive value of USG were found to be 70.0 and 91.3%, respectively, including the case in which a parathyroid lesion was located at the left posterior of the manubrium sterni, an area that is difficult to localize by USG, similar to the study above.

Tc99m sestamibi SPECT, another first-line imaging modality, has variable sensitivity and PPV in different studies. In one study, sensitivity and PPV of Tc99m sestamibi SPECT in single gland disease were found to be 77 and 85%, respectively [25]. Another study demonstrates that overall sensitivity and positive predictive value for localization of parathyroid lesions were 59.8 and 80%, respectively [26]. In our study, the sensitivity and positive predictive value of Tc 99m sestamibi SPECT scan were determined to be 56.7 and 94.4%, respectively. Although our cases predominantly consist of single gland diseases (29/30), the limited sensitivity of sestamibi Tc 99m sestamibi SPECT scan may be attributable to coexisting thyroid nodules (in 9 patients) and the smaller size of certain lesions, which might reduce Tc 99m sestamibi SPECT scan accuracy.

When two initial imaging modalities (USG and Tc 99m sestamibi SPECT scan) fail to identify parathyroid lesions, 4D CT is one of the alternative imaging modalities. Despite its high sensitivity, radiation exposure, which can lead to an age-dependent higher risk of thyroid cancer, is the main limitation of its use [10, 11]. MRI provides superior soft-tissue resolution with no radiation and iodinated contrast exposure, which might be the imaging option for this reason, especially for younger patients [28].

18-fluorocholine-PET-CT had a sensitivity of 99% for identifying parathyroid lesions in patients with negative or discordant MIBI imaging and neck ultrasound results [31]. Despite its superior accuracy, its high cost and limited availability at all institutions restricts its utilization [8]. 4D MRI could be a better option due to its low cost and greater availability compared to 18-fluorocholine-PET-CT in cases where USG and sestamibi scans fail.

Our study has several limitations. First, the retrospective design limits the ability to draw definitive conclusions regarding causal relationships. Second, the sample size was relatively small, as the study cohort included only patients for whom ultrasonography and Tc-99m sestamibi scintigraphy failed to localize the parathyroid glands, necessitating additional imaging. Additionally, the study included only one case of parathyroid hyperplasia, which prevented a thorough evaluation of 4D MRI’s diagnostic accuracy for multiglandular disease. Finally, the lack of post-surgical long-term outcome data, including recurrence rates and follow-up imaging findings, restricts the assessment of the long-term clinical utility of 4D MRI.

Finally, there is a limited number of studies evaluating the sensitivity of 4D MRI in cases of recurrent or multiglandular parathyroid disease. Comparative studies between 4D MRI and other imaging modalities, such as 4D-CT and 18-fluorocholine PET-CT, are also scarce. Further research is needed to better define the role of 4D MRI in these clinical scenarios.

## Conclusion

Dynamic 4D contrast-enhanced MRI utilizes the hypervascular character of parathyroid lesions [14]. 4D MRI successfully detects parathyroid lesions due to its superior soft tissue resolution and the water-fat separation techniques. It offers excellent anatomic detail and can identify small or ectopic parathyroid gland lesions that cannot be detected by USG and sestamibi scans [8].

In conclusion, 4D MRI can be utilized as a complementary imaging modality to identify parathyroid lesions, particularly in cases where they cannot be accurately determined by USG and/or Tc 99m sestamibi SPECT scan or when results of these imaging modalities are discordant with each other, considering the 96.7% sensitivity of the combined use of all imaging modalities. Moreover, due to the absence of ionizing radiation exposure, MRI can be preferred over other alternative imaging techniques, particularly for young patients, as radiation exposure is one of the main causes of thyroid cancer.

## Data Availability

No datasets were generated or analysed during the current study.
